# The interaction of breath holding and muscle mechanoreflex on cardiovascular responses in breath-hold divers and non-breath-hold divers

**DOI:** 10.1007/s00421-024-05431-4

**Published:** 2024-03-05

**Authors:** Nakamura Nobuhiro, Peng Heng, Hayashi Naoyuki

**Affiliations:** 1 Faculty of Sport Sciences, Waseda University, 2-579-15 Mikajima, Tokorozawa, Saitama 359-1192 Japan; 2 Graduate School of Sport Sciences, Waseda University, Tokorozawa, Saitama Japan

**Keywords:** Muscle mechanoreflex, Apnea, Cardiovascular response

## Abstract

Cardiovascular responses to diving are characterized by two opposing responses: tachycardia resulting from exercise and bradycardia resulting from the apnea. The convergence of bradycardia and tachycardia may determine the cardiovascular responses to diving. The purpose of this study was to investigate the interaction of breath holding and muscle mechanoreflex on cardiovascular responses in breath-hold divers (BHDs) and non-BHDs. We compared the cardiovascular responses to combined apnea and the mechanoreflex in BHDs and non-BHDs. All participants undertook three trials—apnea, passive leg cycling (PLC), and combined trials—for 30 s after rest. Cardiovascular variables were measured continuously. Nine BHD (male:female, 4:5; [means ± SD] age, 35 ± 6 years; height, 168.6 ± 4.6 cm; body mass, 58.4 ± 5.9 kg) and eight non-BHD (male:female, 4:4; [means ± SD] age, 35 ± 7 years; height, 163.9 ± 9.1 cm; body mass, 55.6 ± 7.2 kg) participants were included. Compared to the resting baseline, heart rate (HR) and cardiac output (CO) significantly decreased during the combined trial in the BHD group, while they significantly increased during the combined trials in the non-BHD group (*P* < 0.05). Changes in the HR and CO were significantly lower in the BHD group than in the non-BHD group in the combined trial (*P* < 0.05). These results suggest that bradycardia with apnea in BHDs is prioritized over tachycardia with the mechanoreflex, whereas that in non-BHDs is not. This finding implies that diving training changes the interaction between apnea and the mechanoreflex in cardiovascular control.

## Introduction

Cardiovascular responses to breath holding are characterized by bradycardia and peripheral vasoconstriction (Lemaître et al. 2008). This response plays an important role in preserving the oxygen supply to vital organs such as the heart and brain (Nishiyasu et al. 2012; Vestergaard and Larsson 2019) and cardiovascular responses induced by the breath-holding reflex may extend the maximal apnea time (Ferretti 2001). Breath-hold divers (BHDs) have a greater bradycardia with apnea and longer maximal apnea time than healthy age-matched non-breath-hold divers (non-BHDs) (Joulia et al. 2009; Peng et al. 2022). The greater bradycardic response to apnea in BHDs may be related to the divers swimming deeper and/or for longer without breathing.

Divers simultaneously perform exercise and apnea during competitive free diving. Cardiovascular responses to diving are characterized by two opposing responses: tachycardia resulting from the physical activity and bradycardia resulting from the apnea. The magnitude of these opposing responses in freediving differ between BHDs and non-BHDs (Hoffmann et al. 2005; Tocco et al. 2012; Fico et al. 2022). Previous studies have suggested that bradycardia resulting from the apnea was predominant over cardiovascular responses resulting from dynamic apnea, that is, freediving, in BHDs (Tocco et al. 2012), whereas tachycardia resulting from exercise was predominant in non-BHDs (Hoffmann et al. 2005; Fico et al. 2022). The interaction of the apnea and exercise on the cardiovascular response and the differences in the interaction between BHDs and non-BHDs require elucidation.

Cardiovascular responses to exercise are influenced by the exercise pressor reflex (EPR), a feedback mechanism resulting from receptors in exercising skeletal muscles (Gallagher et al. 2006). The EPR is induced by two stimuli: the mechanoreflex originating from physical changes of exercising muscles (Nóbrega et al. 1994; Gladwell and Coote 2002; Tokizawa et al. 2004b; Drew et al. 2008, 2017; Kruse et al. 2016; Lis et al. 2020; Nakamura et al. 2022) and the metaboreflex originating from chemical changes in muscles (Scherrer et al. 1990; Tokizawa et al. 2004a; Ichinose et al. 2006, 2008; Crisafulli et al. 2015).

No study has investigated the interaction of the mechanoreflex and apnea, though the effect of the metaboreflex on the apnea has been investigated in both BHDs (Di Giacomo et al. 2021) and non-BHDs (Ichinose et al. 2018). The metaboreflex mainly affects peripheral vascular tone during dynamic apnea (Ichinose et al. 2018; Di Giacomo et al. 2021). In turn, the extent of convergence of bradycardia associated with apnea and tachycardia associated with the mechanoreflex is responsible for the cardiac response, as the mechanoreflex in humans primarily contributes to the cardioacceleration during exercise. Thus, the present study aimed to compare the cardiovascular responses in BHDs and non-BHDs to investigate the interaction of the effects of apnea and mechanoreflex on cardiovascular responses. Passive leg cycling (PLC) was used to induce the mechanoreflex (Nóbrega et al. 1994; Lis et al. 2020).

## Materials and mthods

### Participants

We recruited nine BHD (male:female, 4:5; age, 35 ± 6 yr; height, 168.6 ± 4.6 cm; body mass, 58.4 ± 5.9 kg; body mass index [BMI], 20.5 ± 1.2 kg/m^2^; relative body fat, 19.2 ± 5.1; means ± standard deviation [SD]) and eight non-divers (male:female, 4:4; age, 35 ± 7 yr; height, 163.9 ± 9.1 cm; body mass, 55.6 ± 7.2 kg; BMI, 20.6 ± 1.0 kg/m^2^; relative body fat, 24.3 ± 6.3%; means ± SD) in this study. The BHDs had personal best of static apnea ≥ 300 s and dynamic with fins ≥ 100 m or constant weight ≥ 40 m. Non-BHDs had not performed habitual aquatic activities and exercise. None of all participants had hypertension (≤ 140/90 mmHg), were smokers, or had cardiovascular disease or diabetes, as assessed by medical history. All participants received verbal and written explanations of the objectives, measurement techniques, and risks and benefits associated with the study and then provided written informed consent to participate before the start of this study. The purpose, procedures, and risks involved in this study were reviewed and approved by the Human Research Committee of Waseda University (approval No. 2021–286). This study was conducted in accordance with the guidelines of the Declaration of Helsinki (1975).

### Protocol

Participants attended the measurement having abstained from strenuous exercise and alcohol for at least 24 h, caffeine for at least 10 h, and food for at least 3 h.

**Fig. 1:**
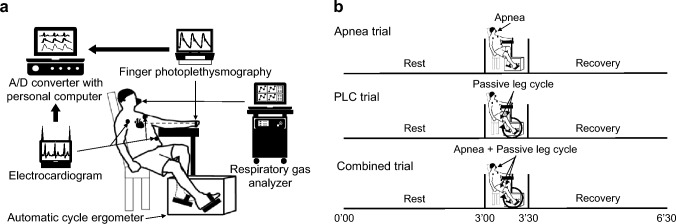
Schematic diagram of the experimental setup (**a**) and overview of the experimental protocol (**b**). Dotted line between heart and finger in panel (**a**) indicates that finger photoplethysmography is located at heart level. *PLC* passive leg cycling

All participants were equipped with measuring devices and rested in a semi-recumbent position for at least 15 min before beginning the protocol (Fig. 1a). The participants undertook the following three trials (Fig. 1b); apnea, passive leg cycling (PLC), and combined trials. The order of the trials was randomized to avoid order effects. Trials were separated by a 3 min rest period. One of the investigators was responsible for the randomization to define the order of three trials. The randomization list was created by function of random number generation using spreadsheet software (Microsoft Excel, Microsoft, WA, USA). The randomized crossover trial was performed in open label.

In the apnea trial, participants performed voluntary apnea with functional residual capacity (FRC) for 30 s. In the PLC trial, passive leg cycling was performed at 60 rpm using automatic cycle ergometer in the semi-recumbent position (AFB3022, Alinco, Osaka, Japan) for 30 s as the mechanoreflex stimulation. Before the PLC trial, the investigator instructed participants to relax to prevent voluntary leg cycling and minimize the unknown effects of other factors such as central command during PLC. No respiratory control was performed during PLC. In the combined trial, participants simultaneously performed apnea and PLC for 30 s. We confirmed breath-holding in the apnea and combined trials using a respiratory gas analyzer (AE-310S, Minato Medical Science Co, Osaka, Japan) with breath-by-breath method. We explained to all participants that FRC is the volume remaining in the lungs after a normal, passive exhalation before the start of the three trials. All participants were instructed to exhale slowly and hold their breath when they reached FRC in apnea and combined trials. All participants familiarized voluntary apnea with FRC and passive leg cycling before beginning the 15 min rest. All measurements were conducted under comfortable laboratory conditions between 14:00 and 18:00.

In an additional experiment on two participants (one BHD and one non-BHD), surface electromyography (EMG) of and vastus lateralis muscles during PLC was recorded to confirm voluntary movement during passive cycle. Signals were amplified by a bioelectric amplifier (MEG-2100, Nihon Kohden, Tokyo, Japan) equipped with input box (JB-210 J, Nihon Kohden, Tokyo, Japan) and obtained at a sampling rate of 1000 Hz through an analog/digital (A/D) converter (PowerLab/16SP, AD Instruments, New South Wales, Australia) and recorded in a device connected to a personal computer (Macbook Pro, Apple, CA, USA).

### Body composition

Body composition was measured using bioelectrical impedance analysis (InBody 720; InBody Japan Inc., Tokyo, Japan) with the participant in the upright position.

### Cardiovascular variables

The HR and beat-to-beat arterial blood pressure waveforms were monitored using a three-lead electrocardiogram (ECG) (BSM-2401, Nihon Kohden, Tokyo, Japan) and finger photoplethysmography (Finometer MIDI, Finapres Medical Systems, Amsterdam, The Netherlands), respectively. The probe of the latter was attached to the middle finger of the left hand which was located at heart level (Fig. 1b). Stroke volume (SV) was calculated based on the obtained arterial blood pressure waveform using the model flow method (Wesseling et al. 1993), which incorporates age, height, and body mass, and simulates aortic flow waveforms from an arterial pressure signal using a non-linear three-element model of the aortic input impedance (Beatscope, version 1.1, Finapres Medical Systems, Amsterdam, The Netherlands). CO and total peripheral resistance (TPR) were then calculated as SV × HR and mean arterial pressure (MAP) / CO, respectively. All hemodynamic measurements at rest were determined by averaging the values during the last 15 s before the start of each trial. All hemodynamic data were also averaged per 5 s during each trial. The recovery data for 60 s were also determined by averaging the values during the last 15 s.

### Heart rate variability

We calculated the HR variability (HRV) to evaluate the cardiac parasympathetic activity. Previous study has reported that the cardiac parasympathetic activity was reflected by the root mean square of standard deviation of R-R intervals (RMSSD) from short-term variation of HR (Task Force of the European Society of Cardiology and the North American Society of Pacing and Electrophysiology 1996). The ECG waveform was obtained at a sampling rate of 1000 Hz through an A/D converter (PowerLab/16SP, AD Instruments, New South Wales, Australia) and recorded in a device connected to a personal computer (Macbook Pro, Apple, CA, USA). Then, the RMSSD were calculated using an analysis software (LabChart8 & MLS 370, AD Instruments, New South Wales, Australia). The RMSSD analysis was conducted by using the last 30 s before the start of trials for the value at rest and the whole 30 s of the trial period.

### Statistical analysis

All data are expressed as the mean ± SD. Statistical analyses were performed using IBM SPSS Statistics for Windows version 27.0 (IBM Corp., Armonk, NY, USA). The effects of time and groups were examined using a two-way repeated-measures ANOVA (time × groups). Significant *F* values were analyzed using Bonferroni’s post hoc test. The changes (Δ) in the HR and CO were calculated as the difference between the 30 s into the trial and the baseline. The mean differences in the characteristics of the participants and the ΔHR and ΔCO between the two groups were examined using the Student’s unpaired *t* test. The sum of the changes in the apnea and PLC trials was calculated to provide an estimated combined response to compare with the actual response in the combined trial: estimated versus actual responses of convergence of the two inputs from apnea and mechanoreflex. In all of the analyses, the level of significance for all comparisons was set at *P* < 0.05.

## Results

One participant in the BHD group was excluded from this analysis because their ABP waveform was not successfully measured. The results were obtained from the remaining eight participants in the BHD group.

**Fig. 2:**
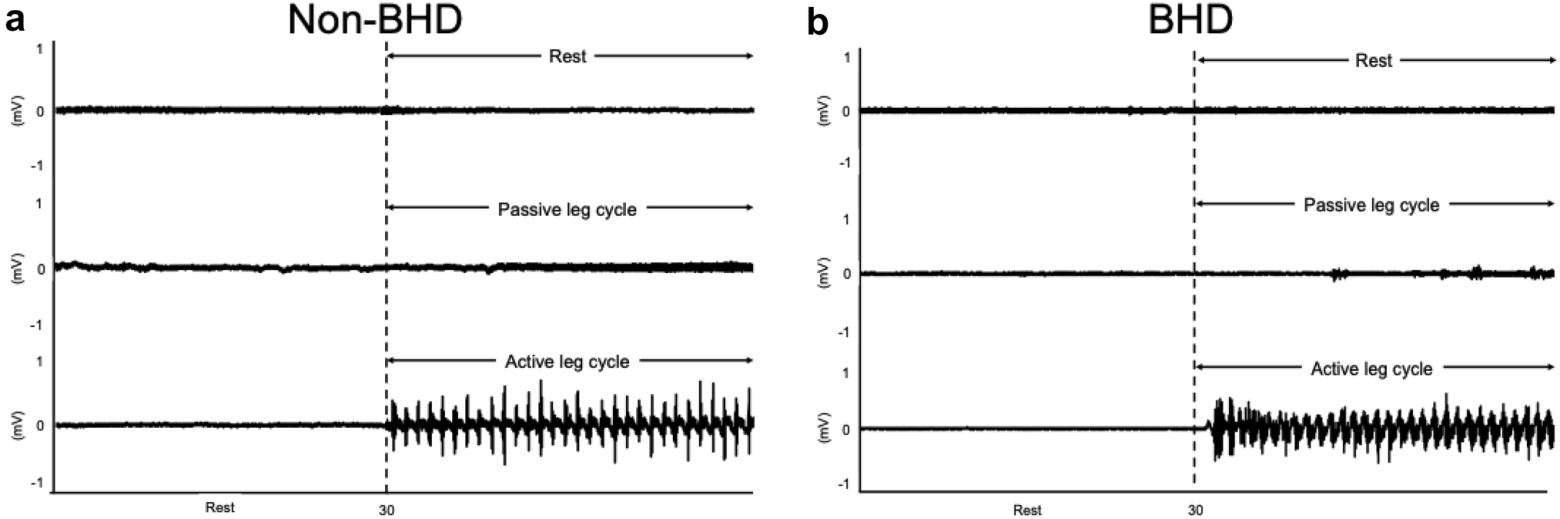
Electromyography recordings of vastus lateralis muscle during passive leg cycling in two subjects; non-BHD (**a**) and BHD (**b**). *BHD*;= breath-holding divers

EMG of vastus lateralis muscles showed no obvious activities in the muscle (Fig. 2).

### Characteristics of the participants

Table 1 presents the characteristics of the participants. No significant difference was observed in all characteristics of the participants.

**Table 1:** Characteristics of the breath-hold diver and non-breath-hold diver groups

Variables	Non-BHD (n = 8)			BHD (n = 8)			*P* value
Age, yrs	35	±	7	35	±	6	0.879
Height, cm	164	±	9	169	±	5	0.197
Body mass, kg	56	±	7	58	±	6	0.392
BMI, kg/m^2^	21	±	1	21	±	1	0.888
Relative fat, %	24.3	±	6.3	19.2	±	5.1	0.084
Personal best STA, s				353	±	45	
Personal best DYN, m				168	±	37	
Personal best CWT, m				53	±	23	

Values are presented as mean ± standard deviation. *BMI* body mass index; *BHD* breath-hold diver; *STA* static apnea; *DYN* dynamic with fins; *CWT* constant weight

### Cardiovascular responses

**Fig. 3:**
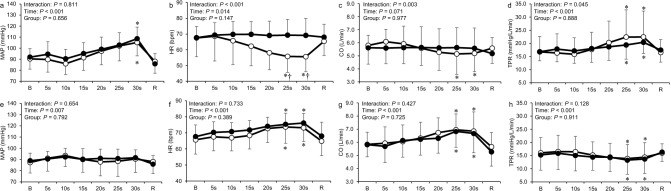
Time course of mean arterial pressure, cardiac output, heart rate, and total peripheral resistance during the apnea (**a**–**d**) and passive leg cycling (**e**–**h**) trials in the non-BHD (●) and BHD (○) groups. *B* baseline; *R* recovery; *CO* cardiac output; *HR* heart rate; *MAP* mean arterial pressure; *TPR* total peripheral resistance. Data are presented as mean ± standard deviation. *Significantly different (*P* < 0.05) compared with baseline. ^†^Significantly different (*P* < 0.05) compared with non-BHD group

Figure 3 shows the time course of all cardiovascular responses to the apnea and PLC trials compared between the two groups. Two-way analysis of variance revealed a significant interaction between time and group on HR, CO, and TPR in the apnea trial (*P* < 0.05; Fig. 3b, c, and d). The HR and CO during the apnea trial in the BHD group decreased significantly at 25 and 30 s (*P* < 0.05). The TPR during the apnea trial in both groups increased significantly at 25 and 30 s (*P* < 0.05). The HR during the apnea trial was significantly lower in the BHD group than in the non-BHD group at 25 and 30 s (*P* < 0.05). In the PLC trial, the HR and CO in both groups increased significantly at 25 and 30 s (*P* < 0.05; Fig. 3f and g), while the TPR decreased significantly at 25 and 30 s (*P* < 0.05; Fig. 3h).

**Fig. 4:**
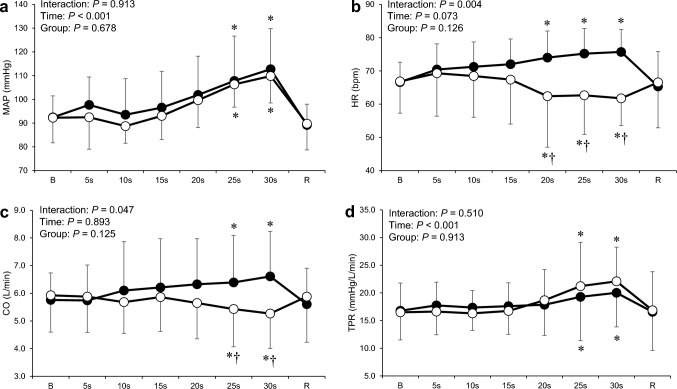
Time course of mean arterial pressure (**a**), heart rate (**b**), cardiac output (**c**), and total peripheral resistance (**d**) during the combined trial in the non-BHD (●) and BHD (○) groups. *B* baseline; *R* recovery; *CO* cardiac output; *HR* heart rate; *MAP* mean arterial pressure; *TPR* total peripheral resistance. Data are presented as mean ± standard deviation. *Significantly different (*P* < 0.05) compared with baseline. ^†^Significantly different (*P* < 0.05) compared with non-BHD group

Figure 4 shows the cardiovascular responses to the combined trials in the two groups. Two-way ANOVA revealed significant interactions between time and group for HR and CO (*P* < 0.05; Fig. 4b and c). A significant reduction in HR during the combined trial was observed at 20 to 30 s compared with that at baseline in the BHD group, whereas an increase in HR in the non-BHD group at the same time point (*P* < 0.05); this indicated a significantly different response between the groups (*P* < 0.05). The CO during the combined trial decreased significantly at 25 and 30 s (*P* < 0.05), whereas an increase in CO in the non-BHD group at the same time point (*P* < 0.05). The CO during the combined trial was significantly lower in the BHD group than in the non-BHD group at 25 and 30 s (*P* < 0.05). The MAP and TPR during the combined trial in both groups increased significantly at 25 and 30 s (*P* < 0.05; Fig. 4a and d).

### Changes in RMSSD

Table 2 shows the RMSSD for all trials. No significant differences were observed between the two groups at baseline. Two-way ANOVA revealed a significant interaction between time and group on RMSSD during the apnea and combined trials (*P* < 0.05). A significant increase in the RMSSD was observed during the apnea and combined trials in the BHD group. The RMSSD during the apnea and combined trials were greater in the BHD group than non-BHD group (*P* < 0.05). A significant reduction in the RMSSD was also observed during the PLC trial (*P* < 0.05).

**Table 2:** Responses in RMSSD to all trials

RMSSD, ms^2^	Baseline			During trial			*P* value
Apnea trial							Interaction = 0.032
Non-BHD	40	±	22	54	±	18	Time = 0.118
BHD	44	±	17	72	±	24*^†^	Group = 0.140
PLC trial							Interaction = 0.819
Non-BHD	44	±	17	30	±	8*	Time = 0.022
BHD	46	±	17	31	±	14*	Group = 0.775
Combined trial							Interaction = 0.003
Non-BHD	43	±	21	30	±	19*	Time = 0.051
BHD	42	±	21	63	±	23*^†^	Group = 0.140

Values are presented as mean ± standard deviation. *BHD* breath-hold diver; *RMSSD* root mean square of standard deviation of R-R intervals; *Significantly different (*P* < 0.05) compared with baseline. ^†^Significantly different (*P* < 0.05) compared with non-BHD group

### Changes in heart rate and cardiac output

**Fig. 5:**
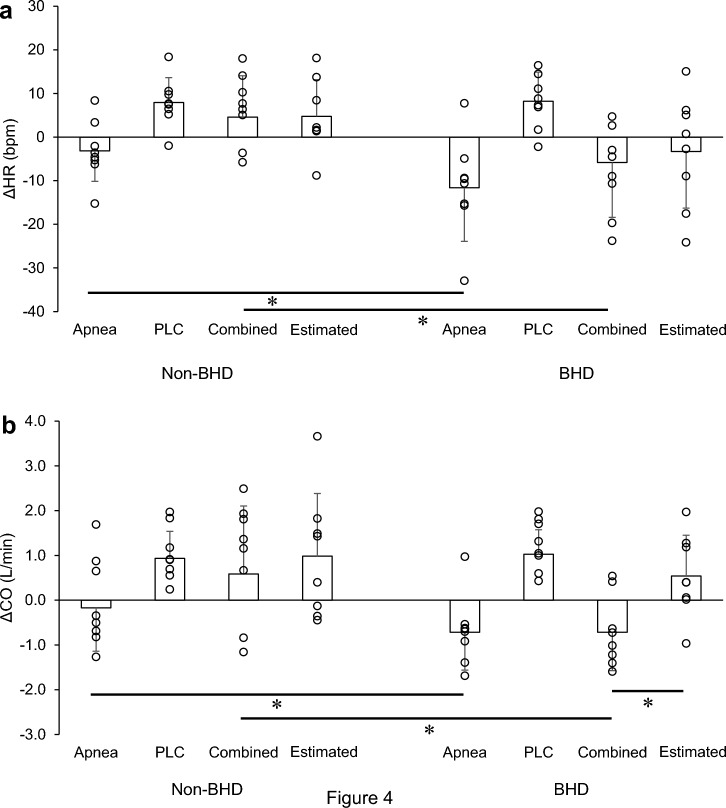
Changes in heart rate (**a**) and cardiac output (**b**) in the apnea, passive leg cycling, and combined trials and the estimated combined trial in the two groups. These data represent individual changes that are underrepresented in Table 2 and Fig. 2. Jitter plots in each bar show individual’s data. *CO* cardiac output; *HR* heart rate; *PLC* passive leg cycling. Data are presented as mean ± standard deviation. *Significantly different (*P* < 0.05)

The ΔHR and ΔCO responses are shown in Fig. 5. The ΔHR and ΔCO were significantly lower in the BHD group than in the non-BHD group in the apnea and combined trials (*P* < 0.05). The estimated responses tended to be positive. In the combined trial, ΔCO was significantly greater in the estimated response than in the actual response (*P* < 0.05; Fig. 5b).

## Discussion

The major findings in the present study were that, during the combined trial, BHDs showed reductions in their HR and CO, whereas non-BHDs showed increases in these measurements. In the BHD group, ΔHR and ΔCO of the combined trial were similar to those in the apnea trial but not the PLC trial; in turn, in the non-BHD group, the ΔHR and ΔCO of the combined trial were similar to those in the PLC trial, despite similar responses in the apnea and PLC trials in both groups (Figs. 3, 4 and 5). These results suggest that the bradycardia with apnea is prioritized over tachycardia with mechanoreflex solely in BHDs. ΔHR and ΔCO of the combined trial were similar to the summation of responses in the apnea and PLC trials—that is, estimated ΔHR and ΔCO—in non-BHDs, whereas they were significantly different from estimated ΔHR and ΔCO in BHDs. The specific regulation in BHDs may result from adaptation for diving deeper and/or longer.

### Cardiovascular responses to apnea and passive leg cycling

Divers showed stronger response to apnea than non-divers. HR and CO were lower in the BHD group than in the non-BHD group (Fig. 3b and c). Previous studies have shown that bradycardia and peripheral vasoconstriction are greater in BHDs than in non-BHDs (Joulia et al. 2009; Peng et al. 2022). A strong bradycardia with apnea is considered a consequence of adaptation to diving and it may extend the breath-holding duration (Schagatay and Andersson 1998).

Cardiovascular response to apnea is mediated by the autonomic nervous system and includes vagally mediated bradycardia and simultaneous increases in sympathetic mediated peripheral vasoconstriction (Hayashi et al. 1997). A previous study indicated that apnea increases cardiac vagal activity (Lemaître et al. 2008), a finding which—as the RMSSD of HR is an indicator of cardiac vagal activity (Task Force of the European Society of Cardiology and the North American Society of Pacing and Electrophysiology 1996)—is supported by the present result of a greater RMSSD response in the BHD group.

Increases in HR and CO during the PLC trial indicated successful stimulation of the mechanoreflex in both groups (Figs. 2, 3f and g). The cardiac response to PLC was probably mainly a consequence of mechanoreflex and of cardiac vagal activity since no obvious voluntary muscular activity was observed and reduction of RMSSD was observed in the present study. Gladwell and Coote (2002) have suggested that passive stretch selectivity suppresses the cardiac parasympathetic activity and increases HR in human study.

The mechanism behind the different cardiac responses to apnea in BHDs and non-BHDs in the present study is unclear. We can only speculate about the possible role of cardiovagal baroreflex sensitivity (BRS). Because the arterial baroreceptors primarily sense the deformation of the arterial wall rather than intraarterial pressure changes (Aars 1969), the cardiovagal BRS is correlated with central arterial mechanical properties such as stiffness and compliance where the baroreceptors are located (the carotid artery and the aortic arch) (Monahan et al. 2001; Mattace-Raso et al. 2007). Lifelong Japanese female divers demonstrate greater reduction in arterial stiffness than age-matched non-divers (Tanaka et al. 2016). The study might suggest that long-term apneic training decreases the arterial stiffness. Thus, BHDs might have had lower arterial stiffness and greater cardiovagal BRS than non-BHDs, resulting in greater bradycardia as a pressor response during apnea. Further studies are needed to investigate the mechanism of how long-term apneic training in BHDs could have contributed to the hemodynamic differences noted compared to those in non-BHDs.

MAP and TPR response to apnea and combined trials were similar between groups (Figs. 3a, d, 4a, d). A study suggested that pressor response during apnea is mainly caused by an increase in TPR (Heusser et al. 2009). There was a greater increase in muscle sympathetic nerve activity (MSNA) at the end of apnea with FRC in BHDs than in non-BHDs (Breskovic et al. 2011). The study also indicated that the augmented sympathetic drive during breath holds was related to chemoreflex stress. FRC breath-hold duration (non-BHD group: 27.7 [22.2–33.2] s, BHD group: 60.4 [34.3–86.5] s) was significantly longer in BHDs, resulting in a higher level of chemoreflex stress. Notably, the slope of the change in total MSNA relative to apnea duration was almost identical between groups, confirming that peripheral and central chemoreflex sensitivity is similar in BHDs and non-BHDs (Dujic et al. 2008; Breskovic et al. 2010), as shown previously, and that different sympathetic responses between groups were due to variations in apnea duration. If BHDs performed apnea at the FRC for the same duration as non-BHDs, the TPR and MAP responses may not have been different, as in the present study.

### Cardiovascular responses to a combination of apnea and mechanoreflex

In the combined trial, the bradycardia with apnea was partially retained in the BHD group, whereas tachycardia was solely observed in the non-BHD group (Fig. 4b). Similarly, HR and CO during the combined trial were suppressed in the BHD group but increased in the non-BHD group. The estimated combined responses of ΔCO differed from the actual responses in the combined trial in the BHD group (Fig. 5b). The ΔCO in the combined trial was similar (that is, negative) to that in the apnea trial. These results indicate that the responses are mainly influenced by the inhibitory effect of apnea on the heart solely in BHDs during combined responses.

The difference in the cardiac response to the combined trial between groups was mainly influenced by the response to apnea and/or the combination of PLC and apnea since the response to PLC was similar. The cardiac response of BHDs in the combined trial may indicate vagally mediated bradycardia with apnea in surplus sympathetically mediated tachycardia with PLC. This is partially supported by the RMSSD, which is an index of cardiac parasympathetic activity, that increased in the BHD group in the combined trial while it decreased in the non-BHD group (Table 2). It is unclear the mechanisms of how to decide which to prize in the antagonistic regulation between vagally mediated bradycardia with apnea and sympathetically mediated tachycardia with the mechanoreflex.

## Limitations

This study had some limitations. First, the generalizability of our findings to other populations is limited because the sample size was small. Second, the present study does not reflect actual diving state such as immersed body. Further studies need to investigate the interaction of apnea and mechanoreflex in real diving condition. Third, we did not evaluate muscle activation across all participants and trials. It is important to conform the absence of active leg cycling during PLC. Fourth, although breathing rate and depth influence HRV analysis (Hirsch and Bishop 1981), we did not regulate the respiratory variables; rather, we only assessed short-term RMSSD (30 s). A study showed that recording RMSSD for at least 240 s and 60 s is required to produce good between- and within-day reliability, respectively (Burma et al. 2021). Thus, care must be taken in proving the involvement of parasympathetic activity in the present study. Fifth, we did not monitor diaphragmatic oscillation to evaluate involuntary breathing movements (IBM). The apnea at FRC would have led to stronger hypoxemic/hypercapnic stress, concurrently increasing the likelihood of IBM occurring. Previous studies have reported the influence of IBM on hemodynamics (Palada et al. 2008; Dujic et al. 2009; Stembridge et al. 2017).

## Conclusions

This is the first study to reveal that the bradycardia with apnea in BHDs is prioritized over tachycardia with the mechanoreflex, whereas the non-BHDs mainly reflects a mechanoreflex-induced response. In the discipline of dynamic apnea, this specific regulation in divers may explain their ability to dive for longer and/or deeper.

## Abbreviations

A/D: Analog/digital
ANOVA: Analysis of variance
BHD: Breath-holding divers
BRS: Baroreflex sensitivity
CO: Cardiac output
ECG: Electrocardiogram
EMG: Electromyography
EPR: Exercise pressor reflex
HR: Heart rate
HRV: Heart rate variability
IBM: Involuntary breathing movement
MAP: Mean arterial pressure
MSNA: Muscle sympathetic nerve activity
PLC: Passive leg cycle
RMSSD: Root mean square of standard deviation of R-R intervals
SV: Stroke volume
TPR: Total peripheral resistance

## References

[CR1] Aars H (1969). Relationship between aortic diameter and aortic baroreceptor activity in normal and hypertensive rabbits. Acta Physiol Scand.

[CR2] Breskovic T, Valic Z, Lipp A (2010). Peripheral chemoreflex regulation of sympathetic vasomotor tone in apnea divers. Clin Auton Res.

[CR3] Breskovic T, Steinback CD, Salmanpour A (2011). Recruitment pattern of sympathetic neurons during breath-holding at different lung volumes in apnea divers and controls. Auton Neurosci.

[CR4] Burma JS, Graver S, Miutz LN (2021). The validity and reliability of ultra-short-term heart rate variability parameters and the influence of physiological covariates. J Appl Physiol.

[CR5] Crisafulli A, Marongiu E, Ogoh S (2015). Cardiovascular reflexes activity and their interaction during exercise exercise : general review and functions. BioMed Res Int.

[CR6] Di Giacomo A, Ghiani GM, Todde F, Tocco F (2021). Cardiovascular responses to simultaneous diving and muscle metaboreflex activation. Front Physiol.

[CR7] Drew RC, Bell MPD, White MJ (2008). Modulation of spontaneous baroreflex control of heart rate and indexes of vagal tone by passive calf muscle stretch during graded metaboreflex activation in humans. J Appl Physiol.

[CR8] Drew RC, Blaha CA, Herr MD (2017). Muscle mechanoreflex activation via passive calf stretch causes renal vasoconstriction in healthy humans. Am J Physiol Regul Integr Comp Physiol.

[CR9] Dujic Z, Ivancev V, Heusser K (2008). Central chemoreflex sensitivity and sympathetic neural outflow in elite breath-hold divers. J Appl Physiol.

[CR10] Dujic Z, Uglesic L, Breskovic T (2009). Involuntary breathing movements improve cerebral oxygenation during apnea struggle phase in elite divers. J Appl Physiol.

[CR11] Ferretti G (2001). Extreme human breath-hold diving. Eur J Appl Physiol.

[CR12] Fico BG, Alhalimi TA, Tanaka H (2022). Vascular responses to simulated breath-hold diving involving multiple reflexes. Am J Physiol Regul Integr Comp Physiol.

[CR13] Gallagher KM, Fadel PJ, Smith SA (2006). The interaction of central command and the exercise pressor reflex in mediating baroreflex resetting during exercise in humans. Exp Physiol.

[CR14] Gladwell VF, Coote JH (2002). Heart rate at the onset of muscle contraction and during passive muscle stretch in humans: a role for mechanoreceptors. J Physiol.

[CR15] Hayashi N, Ishihara M, Tanaka A (1997). Face immersion increases vagal activity as assessed by heart rate variability. Eur J Appl Physiol Occup Physiol.

[CR16] Heusser K, Dzamonja G, Tank J (2009). Cardiovascular regulation during apnea in elite divers. Hypertension.

[CR17] Hirsch JA, Bishop B (1981). Respiratory sinus arrhythmia in humans: How breathing pattern modulates heart rate. Am J Physiol.

[CR18] Hoffmann U, Smerecnik M, Leyk D, Essfeld O (2005). Cardiovascular responses to apnea during dynamic exercise. Int J Sports Med.

[CR19] Ichinose M, Saito M, Kondo N, Nishiyasu T (2006). Time-dependent modulation of arterial baroreflex control of muscle sympathetic nerve activity during isometric exercise in humans. Am J Physiol Heart Circ Physiol.

[CR20] Ichinose M, Saito M, Fujii N (2008). Modulation of the control of muscle sympathetic nerve activity during incremental leg cycling. J Physiol.

[CR21] Ichinose M, Matsumoto M, Fujii N (2018). Voluntary apnea during dynamic exercise activates the muscle metaboreflex in humans. Am J Physiol Heart Circ Physiol.

[CR22] Joulia F, Lemaitre F, Fontanari P (2009). Circulatory effects of apnoea in elite breath-hold divers. Acta Physiol.

[CR23] Kruse NT, Silette CR, Scheuermann BW (2016). Influence of passive stretch on muscle blood flow, oxygenation and central cardiovascular responses in healthy young males. Am J Physiol Heart Circ Physiol.

[CR24] Lemaître F, Buchheit M, Joulia F (2008). Static apnea effect on heart rate and its variability in elite breath-hold divers. Aviat Space Environ Med.

[CR25] Lis A, Łopusiewicz W, Piepoli MF (2020). Passive bilateral leg cycling with concomitant regional circulatory occlusion for testing mechanoreflex–metaboreflex interactions in humans. Clin Auton Res.

[CR26] Mattace-Raso FUS, Van Den Meiracker AH, Bos WJ (2007). Arterial stiffness, cardiovagal baroreflex sensitivity and postural blood pressure changes in older adults: The Rotterdam Study. J Hypertens.

[CR27] Monahan KD, Dinenno FA, Seals DR (2001). Age-associated changes in cardiovagal baroreflex sensitivity are related to central arterial compliance. Am J Physiol-Heart Circ Physiol.

[CR28] Nakamura N, Ikeda N, Heng P, Muraoka I (2022). Muscle stiffening is associated with muscle mechanoreflex-mediated cardioacceleration. Eur J Appl Physiol.

[CR29] Nishiyasu T, Tsukamoto R, Kawai K (2012). Relationships between the extent of apnea-induced bradycardia and the vascular response in the arm and leg during dynamic two-legged knee extension exercise. Am J Physiol Heart Circ Physiol.

[CR30] Nóbrega ACL, Williamson JW, Friedman DB (1994). Cardiovascular responses to active and passive cycling movements. Med Sci Sports Exerc.

[CR31] Palada I, Bakovic D, Valic Z (2008). Restoration of hemodynamics in apnea struggle phase in association with involuntary breathing movements. Respir Physiol Neurobiol.

[CR32] Peng H, Oikawa S, Inai Y (2022). Effects of lung volume and trigeminal nerve stimulation on diving response in breath-hold divers and non-divers. Respir Physiol Neurobiol.

[CR33] Schagatay E, Andersson J (1998). Diving response and apneic time in humans. Undersea Hyperb Med.

[CR34] Scherrer U, Pryor SL, Bertocci LA, Victor RG (1990). Arterial baroreflex buffering of sympathetic activation during exercise-induced elevations in arterial pressure. J Clin Investig.

[CR35] Stembridge M, Hoiland RL, Bain AR (2017). Influence of lung volume on the interaction between cardiac output and cerebrovascular regulation during extreme apnoea. Exp Physiol.

[CR36] Tanaka H, Tomoto T, Kosaki K, Sugawara J (2016). Arterial stiffness of lifelong Japanese female pearl divers. Am J Physiol Regul Integr Comp Physiol.

[CR37] Task Force of the European Society of Cardiology and the North American Society of Pacing and Electrophysiology (1996). Heart rate variability: standards of measurement, physiological interpretation and clinical use. task force of the european society of cardiology and The North American Society of Pacing and Electrophysiology. Circulation.

[CR38] Tocco F, Crisafulli A, Melis F (2012). Cardiovascular adjustments in breath-hold diving: Comparison between divers and non-divers in simulated dynamic apnoea. Eur J Appl Physiol.

[CR39] Tokizawa K, Mizuno M, Nakamura Y, Muraoka I (2004). Venous occlusion to the lower limb attenuates vasoconstriction in the nonexercised limb during posthandgrip muscle ischemia. J Appl Physiol.

[CR40] Tokizawa K, Mizuno M, Nakamura Y, Muraoka I (2004). Passive triceps surae stretch inhibits vasoconstriction in the nonexercised limb during posthandgrip muscle ischemia. J Appl Physiol.

[CR41] Vestergaard MB, Larsson HBW (2019). Cerebral metabolism and vascular reactivity during breath-hold and hypoxic challenge in freedivers and healthy controls. J Cereb Blood Flow Metab.

[CR42] Wesseling KH, Jansen JRC, Settels JJ, Schreuder JJ (1993). Computation of aortic flow from pressure in humans using a nonlinear, three-element model. J Appl Physiol.

